# Mine Gas Time-Series Data Prediction and Fluctuation Monitoring Method Based on Decomposition-Enhanced Cross-Graph Forecasting and Anomaly Finding

**DOI:** 10.3390/s25227014

**Published:** 2025-11-17

**Authors:** Linyu Yuan

**Affiliations:** College of Safety Science and Technology, Xi’an University of Science and Technology, Xi’an 710077, China; 23403070332@stu.xust.edu.cn

**Keywords:** gas time-series prediction, Decomposition-Enhanced Cross-Graph, Multi-Variate Variational Mode Decomposition, Cross Interaction Refinement Graph Neural Network, Pruned Exact Linear Time change-point detection, Shapley Additive Explanations interpretability analysis

## Abstract

Gas disasters in coal mines are the principal constraint on safe operations; accordingly, accurate gas time-series forecasting and real-time fluctuation monitoring are essential for prevention and early warning. A method termed Decomposition-Enhanced Cross-Graph Forecasting and Anomaly Finding is proposed. The Multi-Variate Variational Mode Decomposition (MVMD) algorithm is refined by integrating wavelet denoising with an Entropy Weight Method (EWM) multi-index scheme (seven indicators, including SNR and PSNR; weight-solver error ≤ 0.001, defined as the maximum absolute change between successive weight vectors in the entropy-weight iteration). Through this optimisation, the decomposition parameters are selected as K = 4 (modes) and α = 1000, yielding effective noise reduction on 83,970 multi-channel records from longwall faces; after joint denoising, SSIM reaches 0.9849, representing an improvement of 0.5%–18.7% over standalone wavelet denoising. An interpretable Cross Interaction Refinement Graph Neural Network (CrossGNN) is then constructed. Shapley analysis is employed to quantify feature contributions; the m1t2 gas component attains a SHAP value of 0.025, which is 5.8× that of the wind-speed sensor. For multi-timestep prediction (T0–T2), the model achieves MAE = 0.008705754 and MSE = 0.000242083, which are 8.7% and 12.7% lower, respectively, than those of STGNN and MTGNN. For fluctuation detection, Pruned Exact Linear Time (PELT) with minimum segment length L_min = 58 is combined with a circular block bootstrap test to identify sudden-growth and high-fluctuation segments while controlling FDR = 0.10. Hasse diagrams are further used to elucidate dominance relations among components (e.g., m3t3, the third decomposed component of the T2 gas sensor). Field data analyses substantiate the effectiveness of the approach and provide technical guidance for the intellectualisation of coal-mine safety management.

## 1. Introduction

With the rapid iteration of machine learning models, their application scenarios in the engineering field continue to expand. During the critical stage of the coal mining industry’s transformation towards unmanned and intelligent operations, this technology not only provides underlying support for breakthroughs in core technologies but also fosters new research directions and industrial development paths. In mining operations in areas with high geological disaster risks, gas management remains a long-standing core challenge. Its characteristics of high management difficulty and potential risks not only severely restrict the improvement of mining production efficiency but also pose a direct threat to the life safety of workers and the regional ecological environment. In recent years, research on cutting-edge technologies such as intelligent operation systems and intelligent monitoring systems in the field of mining engineering has been continuously deepened. New algorithms with autonomous decision-making and real-time perception capabilities—such as gas concentration prediction algorithms based on deep learning and multi-source monitoring data fusion algorithms—along with engineering and technological innovations including intelligent gas drainage equipment and dynamic transparent gas geology, are gradually being integrated into the coal mine safety production system. These technological innovations not only provide key support for solving the dilemma of gas management in high geological disaster-risk mining areas and building an efficient safety management and control model but also accelerate the transformation of traditional mining production towards intelligence, refinement, and safety [1]. Currently, scholars have conducted extensive and in-depth research around the above directions, forming rich theoretical and applied achievements.

In the field of signal modal decomposition, mainstream algorithms include Empirical Mode Decomposition (EMD), Complete Ensemble Empirical Mode Decomposition with Adaptive Noise (CEEMDAN), Variational Mode Decomposition (VMD), and Improved Complete Ensemble Empirical Mode Decomposition with Adaptive Noise (ICEEMDAN). Ali et al. [2] used EMD to decompose stock data into multiple components and residuals and constructed a Long Short-Term Memory (LSTM) network using sub-components to predict non-linear and complex financial time-series data, thereby improving the prediction accuracy of complex stock markets. Karijadi et al. [3] employed CEEMDAN to decompose raw energy consumption data into multiple components: The highest-frequency component was input into a Random Forest (RF) for prediction, while the remaining components were predicted using LSTM, achieving excellent prediction results. Liu et al. [4] adopted VMD to reduce the volatility of wind speed sequences and demonstrated that the combined model of VMD, Convolutional Neural Network (CNN), and Gated Recurrent Unit (GRU) has higher prediction accuracy than other single wind power prediction models. To improve the prediction accuracy of gold prices, Liang et al. [5] used ICEEMDAN to decompose the data into components of different frequencies, and then applied LSTM, CNN, and a Convolutional Block Attention Module (CBAM) for joint prediction of all sub-components. All the above studies adopted the approach of “signal decomposition first, then prediction”, and achieved better prediction performance. Therefore, this paper innovatively adopts a framework that integrates the Multi-Variate Variational Mode Decomposition (MVMD) module with a time-series model.

In the field of spatio-temporally correlated time-series prediction models, Fan et al. [6] proposed a Temporal Convolutional Network (TCN) prediction model based on a parallel spatio-temporal attention mechanism, which shortens the training time while improving accuracy. To achieve accurate prediction of power generation in wind farm clusters, Zhang et al. [7] considered the spatio-temporal factors affecting wind power generation and constructed a hybrid model architecture combining CNN and LSTM. Wang et al. [8] proposed a Multi-Component Correlation-Aware Spatio-Temporal Graph Convolutional Network (MC-STGCN), realising accurate multi-scale traffic flow prediction with multivariate correlation awareness. Ding et al. [9] proposed a Multi-Modal Spatio-Temporal Graph Attention Network (MST-GAT), which can capture the spatio-temporal relationships between univariate time series of different modalities in equipment data, improving the accuracy of anomaly detection. Wang et al. [10] proposed a Multi-Scale Graph-based Spatio-Temporal Graph Convolutional Network (MG-STGCN), which effectively captures the spatio-temporal dependencies of data and realises efficient prediction of energy consumption in natural gas pipelines.

In the field of time-series model research, significant progress has been made, but the problem of insufficient model interpretability remains prominent. Thus, it is essential to conduct research on Shapley Additive Explanations (Shapley Analysis). Zhang et al. [11] used Shapley values to explain and analyse variable importance for three extreme responses of wind turbines. Agrawal et al. [12] conducted Shapley Analysis to gain insight into the impact of individual data points on prediction models and quantified the influence of different variables on biodiesel yield prediction. Chang et al. [13] integrated Shapley Analysis into the feature selection process of gas prediction models to identify and quantify the contribution of multivariate features to changes in gas concentration at coal mining faces.

In the field of gas prediction, attention has been directed to the external correlation characteristics of gas concentration data. To address the multiplicity of factors influencing gas concentration, a method combining Recursive Feature Elimination with Cross-Validation (RFECV) and Bidirectional Long Short-Term Memory (BiLSTM) was proposed by Lin et al. [14], by which high predictive accuracy was achieved. Rock engineering system theory was applied by Yu et al. [15] to construct a coal-and-gas outburst prediction model based on the membership degree of cloud-drop graphs for outburst indicators, and the model was used to assess outburst risk in coal seams. A correlation matrix of coal-and-gas outburst indicators was constructed using the Hamming-Distance Artificial Intelligence Algorithm (HDAIA) by Ji et al. [16], and favourable predictive performance for outburst risk was obtained. As gas emission typically increases markedly prior to outburst disasters, precursors have been interpreted from changes in gas concentration. Prediction of methane concentration is regarded as an important means of reducing its impact on coal-mine safety. A spatio-temporal prediction method for methane concentration based on a Graph Convolutional Network–Crossformer (GCN-Crossformer) was proposed by Wang et al. [17] and was shown to be broadly applicable. An interpretable Ali Baba and the Forty Thieves-Transformer–Support Vector Machine (AFT-Transformer-SVM) model was proposed by Wang et al. [18], by which high predictive accuracy for gas emission was achieved. AMS data were used by Juan Díaz et al. [19] to construct a mine-gas forecasting model via an ARIMA approach; it was found that the gas characteristics conformed well to time-series behaviour. A neuro-fuzzy model was employed by Jarosław Brodny et al. [20], with the MHI parameter as the evaluation index, to determine short-term methane susceptibility, thereby enabling effective control in specific mining areas. Artificial neural networks were adopted by Magdalena Tutak et al. [21] to predict methane concentrations at specified points on mine faces, and successful predictions at acceptable accuracy levels were reported for selected extraction points. The shortcomings of traditional gas monitoring and early-warning methods have, to some extent, been mitigated by these studies. The above studies have to some extent made up for the shortcomings of traditional gas monitoring and early warning methods.

To solve the problems of incomplete multi-channel noise suppression, insufficient interpretability of prediction models, and reliance on manual experience for fluctuation monitoring in traditional gas time-series data processing, this paper, based on sorting out and drawing on previous research achievements in fields such as signal modal decomposition, spatio-temporal graph prediction, and change-point detection, proposes an integrated gas time-series data prediction and fluctuation monitoring scheme based on Decomposition-Enhanced Cross-Graph Forecasting and Anomaly Finding. The specific technical route is as follows: Firstly, based on on-site gas monitoring data, the multi-channel multi-modal automatic decomposition method is improved through a combined strategy of “wavelet denoising + information entropy”, and an interpretable Cross Interaction Refinement Graph Neural Network (CrossGNN) is developed to construct a multi-sensor gas data prediction model that can accurately capture data correlation features. Secondly, the Pruned Exact Linear Time (PELT) change-point detection algorithm is introduced to automatically determine the segmentation ratio of time-series data, and a circular block Bootstrap method is used to design a time-series resampling scheme, thereby preserving the intrinsic correlation structure of the original time series. Finally, multiple hypothesis testing is used to control the False Discovery Rate (FDR), ultimately realising segmented fluctuation analysis and sudden increase state monitoring of gas data.

## 2. Materials and Methods

**Figure 1:**
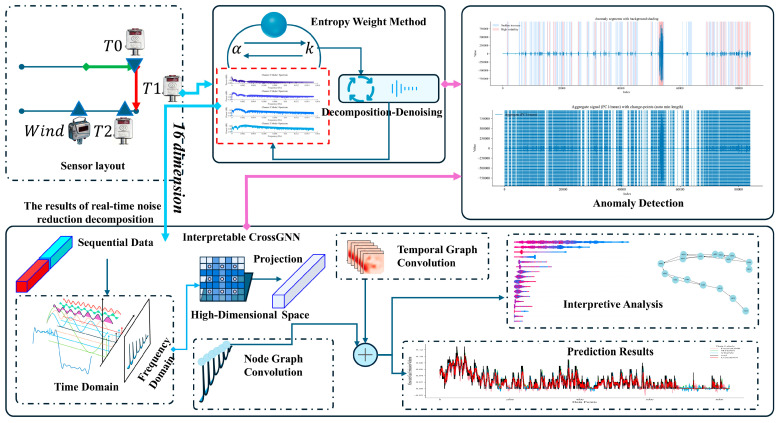
Overall framework diagram of the model.

Figure 1 presents the overall framework of the proposed method. The framework encompasses sensor deployment, data pre-processing, interpretable prediction, and anomaly detection, thereby constituting a complete workflow for gas time-series processing and analysis. First, in the sensor-deployment stage, T0, T1, and T2 gas sensors, together with a wind-speed sensor, are deployed to acquire multi-source gas-related data. Next, the Entropy Weight Method (EWM) is employed to optimise the key parameters (e.g., α and K) of the decomposition–denoising procedure, thereby enabling effective noise suppression and modal decomposition of multi-channel gas time-series data and providing high-quality inputs for downstream processing. On the basis of the decomposed data, inputs are fed to the CrossGNN model. For sequential data, features are first analysed in the time and frequency domains; the data are then mapped to a high-dimensional space and processed by an interpretable Cross-Interaction Refinement Graph Neural Network (interpretable CrossGNN) that incorporates node graph convolution and temporal graph convolution. These components capture, respectively, inter-sensor correlations and temporal dependencies, thereby enabling accurate prediction of gas concentration and the generation of prediction outputs. In parallel, an interpretability module quantifies the contribution of each feature to the predictions, thereby enhancing model transparency. Finally, on the basis of the processed data, an anomaly-detection module is applied to identify sudden abnormalities in gas signals, providing comprehensive support for coal-mine gas safety monitoring and early warning.

### 2.1. Improved MVMD-Based Gas Sensor Data Decomposition Method

Multi-Variate Variational Mode Decomposition (MVMD) [22], proposed by Naveed ur Rehman et al. in 2019, is a signal decomposition method suitable for multi-channel and multi-modal data. Its objective is to develop an effective signal decomposition approach applicable to multi-channel data, enabling efficient signal decomposition and feature preservation. To maximise the extraction of features from multi-sequence gas sensor data, this paper improves the MVMD method by integrating wavelet denoising and the Entropy Weight Method (EWM) for multi-index comprehensive scoring, thereby realising effective feature extraction and decomposition of multi-sequence gas sensor data. The theoretical framework of the improved MVMD-based gas sensor data decomposition method is as follows.

#### 2.1.1. Definition of Variational Problem

As raw mine-acquired data may contain noise that induces substantial prediction error, MVMD decomposition was applied to the original data to enhance model performance [23].

The goal of MVMD is to minimise the sum of the bandwidths of each modal component while constraining the sum of all modes to be equal to the original multi-channel signal. The mathematical model is given by Equation (1):

(1) $${min}_{\left\{u_{k}\right\},\{\omega_{k}\}}\sum_{k=1}^{K}\sum_{c=1}^{C}{\left\|\partial_{t}[(\delta \left(t\right)+\frac{j}{\pi t})*u_{k}^{c}(t)]e^{-j\omega_{k}t}\right\|}_{2}^{2}$$

The constraint condition is $\begin{array}{cc} \sum_{k=1}^{K}u_{k}^{c}\left(t\right)=y^{c}(t) & \left(c=1,2,\ldots ,C\right) \end{array}$, where: $u_{k}^{c}\left(t\right)\;$denotes the k-th modal component of the c-th channel; $\omega_{k}$ is the centre frequency of the k-th mode; $y^{c}\left(t\right)\;$represents the original signal of the c-th channel; C is the number of channels, and K is the number of modes; $\delta \left(t\right)\;$is the Dirac function, and ∗ denotes convolution; $\partial_{t}\;$is the time derivative, and ${\left\|\cdot \right\|}_{2}^{2}$ denotes the squared $L_{2}$ norm.

#### 2.1.2. Unconstrained Transformation via Lagrangian Function

By introducing a penalty parameter α and Lagrange multipliers $\lambda^{c}(t)$, the constrained optimisation problem is transformed into an unconstrained one, as shown in Equation (2):

(2) $$\begin{array}{ll} L\left(\left\{u_{k}^{c}\right\},\left\{\omega_{k}\right\},\left\{\lambda^{c}\right\}\right) & \\ & =\alpha \sum_{k=1}^{K}\;\sum_{c=1}^{C}\;{∥\partial_{t}\left[\left(\delta (t)+\frac{j}{\pi t})*u_{k}^{c}(t\right)\right]e^{-j\omega_{k}t}∥}_{2}^{2}\\ & +\sum_{c=1}^{C}\;{∥y^{c}(t)-\sum_{k=1}^{K}\;u_{k}^{c}(t)∥}_{2}^{2}+\sum_{c=1}^{C}\;\left\langle \lambda^{c}(t),y^{c}(t)-\sum_{k=1}^{K}\;u_{k}^{c}(t)\right\rangle \end{array}$$

where $\left\langle \cdot ,\cdot \right\rangle$ denotes the inner product.

#### 2.1.3. Modal and Centre Frequency Update via Alternating Direction Method of Multipliers

The modal components and centre frequencies are iteratively updated using Fourier transform (frequency-domain solution). The update formulas for the modal component ${\hat{u}}_{k}^{c}$ and centre frequency $\omega_{k}$ are given as Equations (3) and (4):

(3) $${\hat{u}}_{k}^{c}(\omega )=\frac{{\hat{y}}^{c}(\omega )-\sum_{i\neq k}\;{\hat{u}}_{i}^{c}(\omega )+{\hat{\lambda }}^{c}(\omega )/2}{1+2\alpha (\omega -\omega_{k}{)}^{2}}$$

(4) $$\omega_{k}=\frac{\int_{0}^{\infty }\omega {\left|{\hat{u}}_{k}^{c}(\omega )\right|}^{2}d\omega }{\int_{0}^{\infty }{\left|{\hat{u}}_{k}^{c}(\omega )\right|}^{2}d\omega }$$

### 2.2. EWM-Based Multi-Index Comprehensive Scoring Optimisation Method

#### 2.2.1. Index Preprocessing

Since the parameters K (number of modes) and α (penalty parameter) in MVMD-based multi-channel, multi-modal decomposition are difficult to determine, this section calculates the multi-index comprehensive score using the EWM [24] to select the optimal K and α. First, the indices are preprocessed: They are standardised to eliminate dimensional differences and mapped to the range [0,1].

For the positive index Structural Similarity Index Measure (SSIM), the standardisation formulas are given by Equations (5) and (6):

(5) $$x_{norm}=\frac{x-min(x_{all})}{\max\left(x_{all}\right)-min\left(x_{all}\right)+\epsilon }$$

(6) $$SSIM(s,y)=\frac{\left(2\mu_{s}\mu_{y}+C_{1})\cdot (2\sigma_{sy}+C_{2}\right)}{\left(\mu_{s}^{2}+\mu_{y}^{2}+C_{1})\cdot (\sigma_{s}^{2}+\sigma_{y}^{2}+C_{2}\right)}$$

In Equation (5), ϵ is a minimal value used to avoid division by zero; x denotes the SSIM value corresponding to a specific combination of K and α; $x_{all}$ is the set of SSIM values across all parameter combinations; $min(x_{all})$ and $max(x_{all})$ are the minimum and maximum values of the index across all parameter combinations, respectively.

In Equation (6), s is the denoised signal, and y is the original signal; $\mu_{s}$ and $\mu_{s}$ are the means of signals s and y, respectively; $\sigma_{s}^{2}$ and $\sigma_{y}^{2}$ are the variances of signals s and y, respectively; $\sigma_{sy}$ is the covariance between signals s and y; $C_{1}={\left(K_{1}\cdot L\right)}^{2}$ and $C_{2}={\left(K_{2}\cdot L\right)}^{2}$, where $K_{1}=0.01$ and $K_{2}=0.03$ are empirical values; L is the dynamic range of the signal, defined as the difference between the maximum and minimum values of the signal.

#### 2.2.2. Information Entropy and Weight Calculation

To improve calculation effectiveness, multiple evaluation indices are selected for comprehensive calculation to assess optimisation performance. However, the weight ratio between different parameters is difficult to determine during optimisation; thus, information entropy is used to automatically calculate the weight ratio between variables, achieving optimal MVMD algorithm optimisation. The formulas are shown in Equations (7)–(9):

(7) $$p_{ij}=\frac{x_{norm,ij}}{\sum_{i=1}^{n}\;x_{norm,ij}+\epsilon }$$

Equation (7) calculates the index weight $p_{ij}$ for the MVMD algorithm, where $p_{ij}$ represents the proportion of the standardised value of the i-th parameter combination (e.g., a specific K-α pair) in the j-th index to the sum of standardised values of all parameter combinations for that index. $x_{norm,ij}$ is obtained via data standardisation (based on index values across all parameter combinations), and $p_{ij}$ is the relative proportion of standardised values, determined entirely by the data without manual intervention.

Equation (8) calculates the information entropy $e_{j}$, which measures the “information content” of an index:

(8) $$\begin{array}{cc} e_{j}=-\frac{1}{log(n)}\sum_{i=1}^{n}\;p_{ij}log(p_{ij}) & \left(p_{ij}>0\right) \end{array}$$

In Equation (8), information entropy quantifies the “dispersion degree” of an index: If the values of an index across all parameter combinations are highly similar (e.g., SSIM values of all combinations are close to 0.8), $p_{ij}$ is uniformly distributed, and $e_{j}$ approaches its maximum value (high entropy), indicating weak ability of the index to distinguish between good and bad parameters; if the values of an index across parameter combinations vary significantly (e.g., SSIM fluctuates between 0.5 and 0.9), $p_{ij}$ is unevenly distributed, and $e_{j}$ is small, indicating strong distinguishing ability (high information content) of the index.

$e_{j}\;$is calculated entirely from $p_{ij}$, which is in turn determined by the original index data. The entire process relies only on the statistical properties of the data, requiring no manual judgment.

(9) $$\begin{array}{cc} w_{j}=\frac{1-e_{j}}{\sum_{j=1}^{m}\;(1-e_{j})} & \left(\sum_{j=1}^{m}\;w_{j}=1\right) \end{array}$$

Equation (9) calculates the entropy weight $w_{j}$, which quantifies the “importance” of an index. It follows the principle: “the smaller the information entropy, the larger the weight”. Specifically, if an index j has a small $e_{j}$ (high information content and strong distinguishing ability), $1-e_{j}$ is large, leading to a high weight $w_{j}$; if an index j has a large $e_{j}$ (low information content and weak distinguishing ability), $1-e_{j}$ is small, leading to a low weight $w_{j}$. The selected indices include Signal-to-Noise Ratio (SNR), Peak Signal-to-Noise Ratio (PSNR), SSIM, Mean Absolute Error (MAE), Root Mean Squared Error (RMSE), Pearson correlation coefficient, and spectral kurtosis.

Direct summation of all modes after the original MVMD was found to retain noise components. To reduce redundant noise, relevant modes were automatically selected by computing the Pearson correlation between each mode and the raw signal. The signal was then decomposed via MVMD; effective modes were retained, and three-level soft-threshold wavelet denoising (db4) was applied. By exploiting MVMD’s strength in modal separation and wavelets’ strength in local time-domain denoising, more thorough noise suppression was achieved than with either method alone. On this basis, the acquired data were decomposed and a corresponding scoring scheme was defined.

In order to evaluate denoising and decomposition quality from complementary perspectives, seven indices were adopted in the entropy-weight optimisation: Signal-to-Noise Ratio (SNR), Peak Signal-to-Noise Ratio (PSNR), Structural Similarity Index Measure (SSIM), Mean Absolute Error (MAE), Root Mean Squared Error (RMSE), Pearson correlation coefficient, and spectral kurtosis. Collectively, these indices cover amplitude fidelity and large-error suppression (MAE/RMSE), structural preservation (SSIM), linear association with the original signal (correlation), robustness to impulsive/non-Gaussian components (spectral kurtosis), and overall noise suppression (SNR/PSNR). Because underground gas signals are multi-channel, wide-band and often contaminated by sporadic spikes and non-stationary interference, a single metric is insufficient; the above set reduces metric bias (e.g., high SNR with degraded structure) and improves discriminative power across (K,α) candidates. All indices were standardised to [0,1] (see Equations (5) and (6)), and their data-driven entropy weights $w_{j}$ down-weight poorly discriminative indices while amplifying those that separate good from poor parameter choices; the reported weight computation error (≤0.001) indicates stable weighting under the present dataset.

#### 2.2.3. Comprehensive Score Calculation

The final comprehensive score, Score, is calculated using Equation (10). A higher score indicates a more optimal parameter combination:

(10) $$Score=\sum_{j=1}^{m}\;w_{j}\cdot x_{norm,j}$$

#### 2.2.4. Wavelet Denoising Method

Building on the above model, a denoising model was configured; its theoretical underpinnings are as follows.

(11) $$s(t)=\sum_{j=1}^{J}\;c_{j}+\sum_{j=1}^{J}\;d_{j}$$

Equation (11) decomposes the original signal s(t) into low-frequency approximate components and high-frequency detail components across multiple scales, where J is the number of decomposition levels; $c_{j}$ is the approximate coefficient of the j-th level; and $d_{j}$ is the detail coefficient of the j-th level.

(12) $$d_{j}^{\prime }=c\cdot max(0,|d_{j}|-thr)$$

Equation (12) filters the high-frequency detail coefficients $d_{j}$ to remove small-magnitude coefficients corresponding to noise, where $d_{j}$ is the original high-frequency detail coefficient (containing noise); $d_{j}^{\prime }$ is the processed high-frequency detail coefficient (with noise suppressed); $d_{j}^{\prime }$ is the sign function (preserving the positive/negative direction of the coefficient); thr is the threshold distinguishing noise from the signal; and $max(0,|d_{j}|-thr)$ retains the value of $|d_{j}|-thr$ if |d_j | > thr (for smooth transition) and sets the coefficient to 0 if $\left|d_{j}\right|<thr$ (to remove noise).

The threshold thr is determined via “minimum risk threshold selection”, which automatically calculates the threshold that minimises the risk of estimation error. The simplified formula is given by Equation (13):

(13) $$thr=arg\underset{\lambda }{min}\;E[(\hat{x}-x{)}^{2}]$$

Equation (13) identifies a threshold λ by analyzing the distribution of detail coefficients, such that the Mean Squared Error (MSE) between the denoised signal $\hat{x}$ and the true signal x is minimised. This method requires no manual threshold setting and has strong adaptability.

Finally, wavelet reconstruction is performed based on the above calculations, as shown in Equation (14):

(14) $$s^{\prime }(t)=\sum_{j=1}^{J}\;c_{j}+\sum_{j=1}^{J}\;d_{j}^{\prime }$$

Equation (14) is the wavelet reconstruction formula, which recombines the processed high-frequency detail coefficients $d_{j}^{\prime }$ with the original low-frequency approximate coefficients $c_{j}$ to restore the time-domain signal (i.e., the denoised signal). The db4 wavelet basis is selected for wavelet denoising, which follows a three-step process—“decomposition–thresholding–reconstruction”—to specifically suppress high-frequency noise and separate signal components of different frequencies. When combined with MVMD, MVMD first decomposes the signal into modal components with higher purity; wavelet denoising then performs refined denoising on these components.

This “separation first, purification later” approach achieves efficient denoising for multi-channel, wide-band, noisy signals.

### 2.3. Gas Time-Series Data Prediction and Data Importance Analysis Method

The Cross Interaction Refinement Graph Neural Network (CrossGNN) [25], proposed by Qihe Huang and Lei Shen et al. in 2023, is a method for processing noisy multivariate time-series data. By integrating an adaptive multi-scale identifier and cross-scale Graph Neural Network (GNN), it can accurately extract relevant features from multi-scale time-series data. To deeply analyse the model’s decision-making process and verify the credibility of the results, the Shapley Additive Explanations (Shapley Analysis) method is integrated into the original CrossGNN. By quantifying the contribution of each input feature to the model output, this integration effectively addresses the lack of interpretability in the original method.

#### 2.3.1. GrossGNN Model Architecture

(1) Adaptive Period Identification for Multi-Channel Gas Data

For the input gas time-series data $X\in R^{B\times L\times D}$ (where B is the batch size, L is the sequence length, and D is the feature dimension), the Top-K significant periods are automatically extracted via FFT-based period detection [26], as shown in Equation (15):

(15) $$FFT(X)=F(X)\in C^{\frac{BL}{3}+D}$$

The frequency energy is defined by Equation (16):

(16) $$E[f]=mean(|FFT(X)|,dim=(0,2))\in R^{\frac{L}{2}+1}$$

The periods corresponding to the k frequencies with the highest energy are selected for subsequent calculations, as shown in Equation (17):

(17) P={⌈L/f⌉∣f=top−k(E[f])}∪{1}

(2) Multi-Scale Data Generation

For each period p∈P, a multi-scale sequence is generated via moving-average downsampling, as shown in Equation (18):

(18) $$X^{\left(p\right)}=avgpool(X,kernel=p,stride=p)\in R^{B\times \lfloor L/p\rfloor \times D}$$

Finally, the multi-scale sequences are concatenated and aligned to the maximum scale $L_{max}$, as shown in Equation (19):

(19) $$X_{multi}=concat(\{X^{\left(p\right)}|p\in P\})\in R^{B\times L_{max}\times D}$$

#### 2.3.2. Cross-Scale Graph Neural Network

(1) Feature Dimension Expansion

The data features are mapped to a high-dimensional space, as shown in Equation (20):

(20) $$X_{multi}=concat(\{X^{\left(p\right)}|p\in P\})\in R^{B\times L_{max}\times D}$$

where $W_{proj}\in R^{1\times H}$ is the projection matrix, and H is the hidden dimension.

(2) Temporal Graph Convolution

First, a learnable temporal embedding vector is used to generate an adaptive adjacency matrix, which captures dependencies in the temporal dimension, as shown in Equation (21):

(21) $$A_{t}=softmax\left(ReLU\left(V_{t1}\cdot V_{t2}^{T}\right)⊙M\right)\in R^{L_{max}\times L_{max}}$$

where $V_{t1}$ in $R^{L_{max}\times E_{t}}$ and $V_{t2}\in R^{L_{max}\times E_{t}}$ are temporal embedding vectors 1 and 2, respectively; $M\in R^{L_{max}\times L_{max}}$ is a mask matrix that filters non-critical temporal links and retains adjacent and cross-scale dependencies; ⊙ denotes element-wise multiplication.

Subsequently, a K-order temporal graph convolution is performed to aggregate K-order neighbor information for the high-dimensional embedded features $X_{emb}\in R^{B\times L_{max}\times H}$ (where B is the batch size and H is the hidden dimension), as shown in Equation (22):

(22) $$X_{t}^{\left(K\right)}=concat\left(X_{emb},\sum_{k=1}^{K}\;\left(X_{emb}\cdot A_{t}^{k}\right)\cdot W_{tk}\right)\cdot W_{tfusion}\in R^{B\times L_{max}\times H}$$

where $A_{t}^{k}$ is the k-th power of the temporal adjacency matrix $A_{t}$; $W_{tk}\in R^{H\times H}$ is the k-th order temporal convolution weight; $W_{tfusion}\in R^{2H\times H}$ is the fusion weight, which concatenates the original features with the K-order aggregated features and compresses them to the H-dimensional space; concat(·) denotes the feature concatenation operation.

(3) Node Graph Convolution

A learnable node embedding vector is used to generate an adaptive adjacency matrix, which captures dependencies in the feature dimension (nodes), as shown in Equation (23):

(23) $$A_{n}=softmax\left(ReLU\left(V_{n1}\cdot V_{n2}^{T}\right)⊙M_{n}\right)\in R^{D\times D}$$

where $V_{n1}$ in $R^{D\times E_{n}}$ and $V_{n2}$ in $R^{D\times E_{n}}$ are node embedding vectors 1 and 2, respectively; $M_{n}\in R^{D\times D}$ is a node mask matrix used to retain Top-k critical nodes and suppress negative correlations. Thereafter, the graph convolution operation is implemented, as shown in Equation (24):

(24) $$X_{n}=\sum_{i=0}^{K}\;(A_{n}^{i}\cdot X_{emb}^{T})\cdot W_{n}^{\left(i\right)}\in R^{B\times L_{max}\times H}$$

(4) Feature Fusion and Prediction

The original features and graph convolution outputs are concatenated, and prediction is performed via a linear layer, as shown in Equation (25):

(25) $$\hat{Y}=W_{pred}\cdot concat(X_{emb},X_{t},X_{n})\in R^{B\times T\times 3}$$

where T is the prediction horizon, and $W_{pred}\;$in$\;R^{3H\times 3}$ is the prediction weight.

#### 2.3.3. Shapley Interpretability Analysis

For the model output ${\hat{Y}}_{i}$, the Shapley value of a feature f is defined as its average marginal contribution across all feature subsets, as shown in Equation (26):

(26) Shap(f,Y^i)=1D!∑S⊆F∖{f} (D−1|S|)−1Y^i(S∪{f})−Y^i(S)

where F is the set of all features, and S is a subset excluding f.

Based on the feature importance derived from Shapley values—$Imp\left(f\right)=mean(|Shap(f,{\hat{Y}}_{i})|)$—a partial order relationship is defined, as shown in Equation (27):

(27) $$f_{1}⪯f_{2}⟺Imp(f_{2})\geq Imp(f_{1})\cdot (1+\tau )$$

where τ is a threshold. If this relationship is satisfied, a directed edge from $f_{1}$ to $f_{2}$ is established, forming a Hasse diagram.

### 2.4. Unsupervised Diagnosis Method for Gas Data

For enhanced analysis, an unsupervised gas-fluctuation monitoring approach was adopted. The decomposed data were analysed in detail, and the theoretical foundations are outlined below.

#### 2.4.1. Data Preprocessing

For data decomposed via MVMD, robust standardisation is applied for preprocessing. Missing values are imputed using interpolation combined with forward–backward filling, as shown in Equation (28):

(28) $$x_{t,j}^{\prime }=\frac{x_{t,j}-median(x_{\cdot ,j})}{MAD(x_{\cdot ,j})}$$

where MAD denotes the Median Absolute Deviation.

Subsequently, the minimum segment length $L_{min}$ is automatically determined by integrating the main peak period P (from the frequency domain) and the lag $L_{acf}$ (where the Autocorrelation Function (ACF) first drops below 0.1). The larger value of these two is selected and clamped, as shown in Equation (29):

(29) $$L_{min}=min(max(30,2P,3L_{acf}),max(30,\lfloor N/3\rfloor ))$$

where N is the sample length.

#### 2.4.2. Mean Change-Point Detection After Segmentation

The Pruned Exact Linear Time (PELT) algorithm with the ‘l2’ model is used to detect mean change points in the multivariate matrix X. PELT is re-run on the first-order difference ∆X to capture slope changes, and the change points are merged. During detection, the penalty terms are adaptive to the sample length and dimension, as shown in Equation (30):

(30) $${pen}_{mean}=3\left(logN\right)d\;\;\;\;\;\;\;\;\;\;\;\;{pen}_{diff}=2\left(logN\right)d$$

Subsequently, statistical analysis is performed for each segment i: The multi-column mean $\mu_{i}$; the average of multi-column variances $\sigma_{i}^{2}$; the linear slope $a_{i}$ of the aggregated signal; the mean difference between adjacent segments ${∆}_{i}=\mu_{i}-\mu_{i-1}$. ${\mathrm{pen}}_{\mathrm{mean}}$ is the penalty used when PELT is applied to X (detecting level/mean changes), whereas ${\mathrm{pen}}_{\mathrm{diff}}$ is used when PELT is applied to ΔX (detecting slope/gradient changes). The larger constant in ${\mathrm{pen}}_{\mathrm{mean}}=3(logN)d$ imposes stricter regularisation to avoid over-segmenting mean shifts, while ${\mathrm{pen}}_{\mathrm{diff}}=2(logN)d$ is slightly lighter to retain sensitivity to slope changes; both scale with logN and d to adapt to sample length and dimensionality.

A circular block Bootstrap method (with block length $b\approx N^{1/3}$ is used to generate B correlation-preserving pseudo-samples from the aggregated signal agg, which are used to construct the empirical null distribution for sudden increases and high fluctuations. The studentised mean jump statistic between adjacent segments i−1 and i is calculated on the aggregated signal, as shown in Equation (31):

(31) $$Z_{i}=\frac{{\bar{x}}_{i}-{\bar{x}}_{i-1}}{\sqrt{s_{p}^{2}\left(\frac{1}{n_{i}}+\frac{1}{n_{i-1}}\right)}},S_{p}^{2}=\frac{(n_{i-1}-1)s_{i-1}^{2}+(n_{i}-1)s_{i}^{2}}{n_{i-1}+n_{i}-2}$$

Only right-tail testing is performed for $Z_{i}$. The empirical p-value is derived from the block Bootstrap null distribution $\left\{Z^{*}\right\}$, as shown in Equation (32):

(32) $${\hat{p}}_{i}=\frac{1+|\{z^{*}|z^{*}\geq z_{i}\}|}{1+|\{z^{*}\}|}$$

#### 2.4.3. Significance Testing for High-Fluctuation Segments

The average of multi-column variances within a segment, ${\;\sigma }_{i}^{2}$, is used to measure fluctuation. For each segment length $L_{i}$, sliding windows of the same length are sampled from the Bootstrap samples, and their variances $v^{*}$ are collected to form the null distribution. The right-tail empirical p-value is calculated as in Equation (33):

(33) $${\hat{p}}_{i}=\frac{1+|\{v^{*}|v^{*}\geq \sigma_{i}^{2}\}|}{1+|\{v^{*}\}|}$$

Finally, multiple corrections are applied to the overall results: Sort the set of p-values $p_{k}$ in ascending order; select the largest $k^{*}$ such that $p_{\left(k^{⋆}\right)}\leq \frac{k^{⋆}}{m}q$, where m is the number of tests and q is the False Discovery Rate (FDR) level; and segments i that pass the threshold are labelled as “Sudden increase” (for mean jumps) or “High volatility” (for high variance).

## 3. Results

To achieve accurate analysis of gas features, this paper selects 83,970 sets of coal mining face data from a data centre in Shaanxi Province as core samples, and conducts in-depth research focusing on the construction of gas prediction models and real-time fluctuation monitoring.

### 3.1. Data Decomposition and Preprocessing

**Figure 2:**
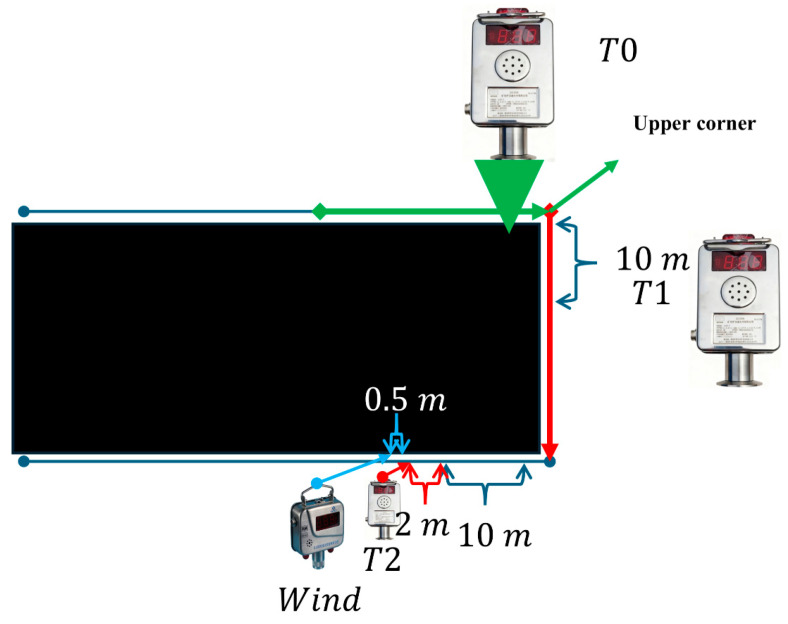
Sensor installation location diagram.

Figure 2 illustrates the sensor installation scheme. A T0 gas laser sensor is installed at the upper corner of the coal-mining face, a T1 gas laser sensor is positioned 12 m from the face along the gate road, and a T2 gas laser sensor is installed 10 m from the return gate road. Laser wind-speed sensors are placed at 0.5-metre intervals. The gas sensors are of model KG9001C (measurement range 0–100% CH_4_; alarm set-point range 0.5–2.5% CH_4_), and the wind-speed sensors are GFC20(A) (measurement range 0.1–20 m·s^−1^, with upper/lower limit alarms). Raw signals are read periodically by an external KXJ127 controller at 0.0333 Hz and uploaded to the data centre. All hyperparameter selections are listed in Table A1, and all core pseudocode is provided in Algorithms A1 and A2.

Green arrows indicate fresh airflow, while red arrows represent contaminated air flow. First, for the original data from the three gas sensors (T0, T1, T2) and the wind-speed sensor, analysis was conducted using the Entropy Weight Method (EWM) combined with the improved Multi-Variate Variational Mode Decomposition (MVMD). All four types of sensors have a sampling frequency of 0.0333 Hz (one sample every 30 s). Wavelet denoising alone was used for comparison to verify the efficiency of the proposed algorithm.

**Figure 3:**
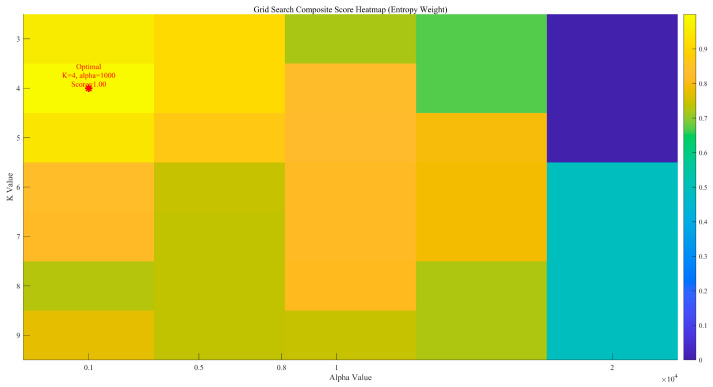
Heatmap of scores calculated by the Entropy Weight Method.

**Figure 4:**
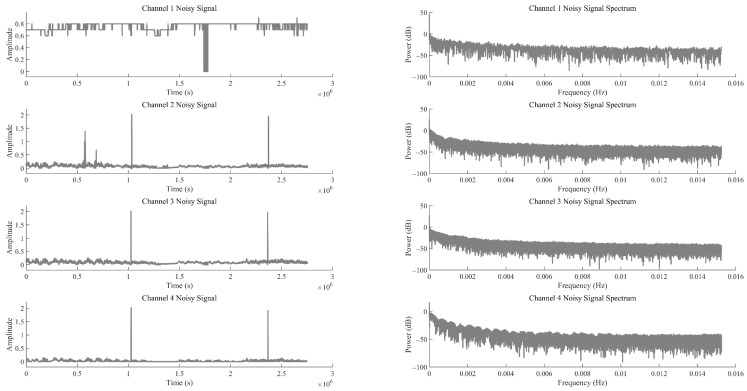
Time-frequency diagrams of noisy signals.

Considering the difficulty in accurately determining the order (parameters K and α) during the decomposition process of the improved MVMD, the EWM was further introduced to optimise the key parameters K (number of decomposition modes) and α (penalty parameter). The optimisation results are shown in Figure 3. It can be seen from Figure 3 that the optimal decomposition order K of the improved MVMD model is 4, and the optimal parameter α is 1000. Figure 4 presents the time-domain waveforms and frequency-domain spectra of the noisy signals from all original channels. Each channel corresponds to two subfigures: the left subfigure shows the amplitude variation of the signal over time, and the right subfigure displays the frequency distribution of the signal. These subfigures are used to preliminarily identify the time-domain and frequency-domain characteristics of noise interference, where Channels 1–4 correspond to data from the T0, T1, and T2 gas sensors, and the wind-speed sensor, respectively.

As shown by the EWM score heatmap in Figure 3, the comprehensive score exhibits a clear optimum around K=4 with α≈1000, accompanied by a locally flat high-score region in its neighbourhood. The position of the red asterisk indicates the location of the optimal parameter selection result. Departures from K=4  reduce the score because too small a mode number merges distinct components (under-decomposition), whereas too large a mode number fragments energy and re-introduces noise (over-decomposition). Likewise, very small α yields insufficient bandwidth control and residual high-frequency noise, while excessively large α over-smooths modes and attenuates useful detail. The superiority of the parameter pair selected from Figure 3 is consistent with Table 1, where the joint (MVMD + wavelet) results show improved PSNR/SSIM/SNR over the single-stage baseline on representative channels, indicating that the chosen K and α strike an effective balance between mode purity and detail retention under the EWM objective.

**Figure 5:**
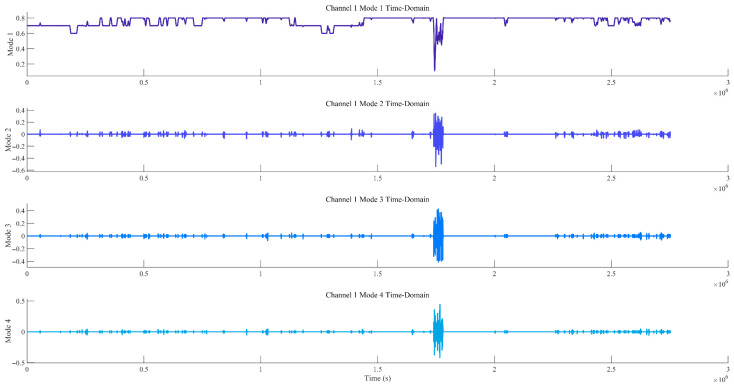
Time-domain diagrams of decomposed modal components for channel 1.

**Figure 6:**
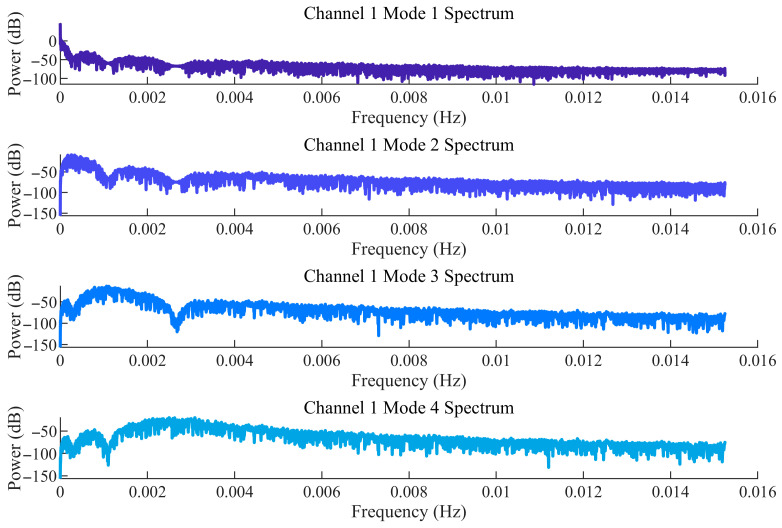
Frequency-domain spectra of modal components for channel 1.

Figure 5 and Figure 6 show the time-domain diagrams and frequency-domain spectra of the modal components of Channel 1, respectively. Figure 5 displays the time-domain waveforms of the modal components of Channel 1 after MVMD decomposition. It can be observed from the figure that the modes exhibit clear temporal variation patterns, and the modes are well decomposed, with distinct features visible after decomposition. Figure 6 presents the frequency-domain spectra of the modal components, showing the frequency distribution of each modal component after decomposition. This helps determine the frequency space corresponding to each mode and assists in identifying noise modes and valid signal modes. Due to the large volume of data, only the results for Channel 1 are presented here.

**Figure 7:**
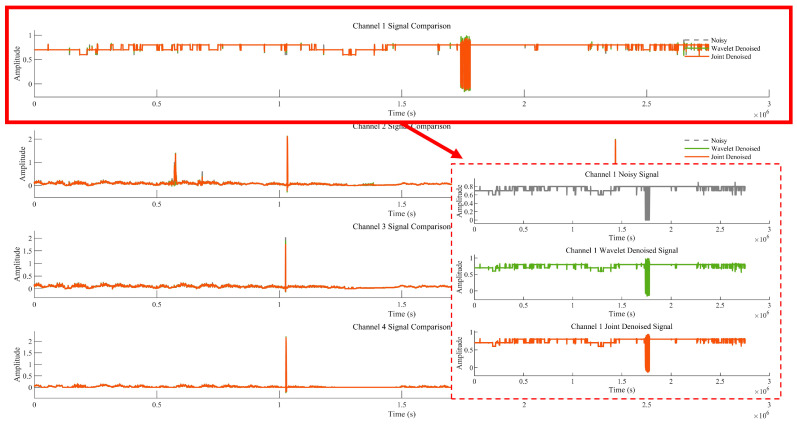
Comparison results of wavelet denoising alone and improved MVMD denoising methods.

**Figure 8:**
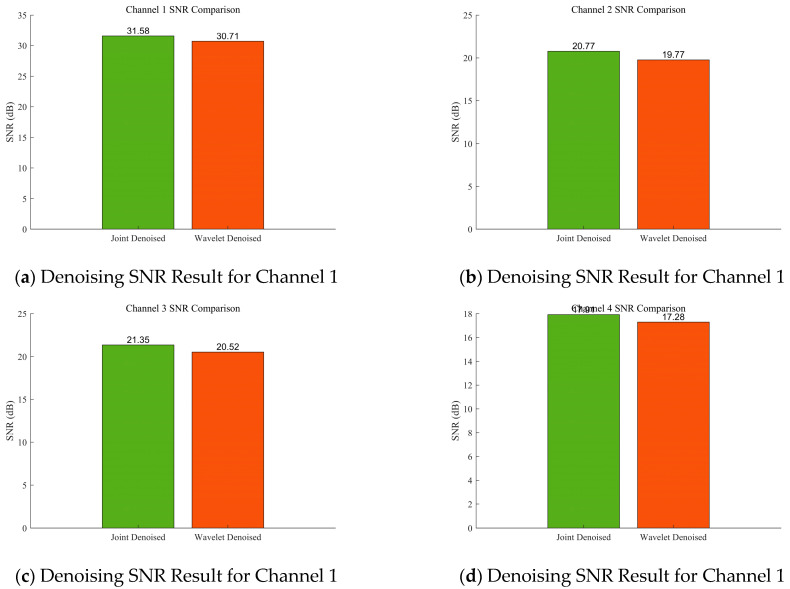
SNR comparison diagram of wavelet denoising alone and improved MVMD denoising methods.

To better analyse the results of the improved MVMD algorithm, Figure 7 and Figure 8 show the comparison between wavelet denoising alone and joint denoising (MVMD + wavelet denoising) results. Figure 7 displays the noisy signal, the result of wavelet denoising alone, and the result of joint denoising: the signal is first decomposed into four components, and wavelet denoising is applied to these components either individually (wavelet denoising alone) or in combination with MVMD (joint denoising) for comparison. Figure 8 shows the Signal-to-Noise Ratio (SNR) values of the denoising results—SNR is used to evaluate the performance difference between algorithms [27], with a higher SNR indicating a better denoising effect. The red area is the clear version corresponding to the Channel Noisy Signal.

Table 1 presents the corresponding calculation results, where Peak Signal-to-Noise Ratio (PSNR): A higher value indicates a better signal quality; Structural Similarity Index Measure (SSIM): Evaluates signal similarity from three aspects (brightness, contrast, and structure), with a value closer to 1 indicating a more complete denoised signal; Mean Absolute Error (MAE): Calculates the average absolute deviation between the denoised signal and the original signal, with a smaller value indicating a smaller difference between the two; Root Mean Squared Error (RMSE): Amplifies the impact of large errors, with a smaller value indicating better denoising performance; Correlation Coefficient: Measures the linear correlation between the original and denoised signals, with a value closer to 1 indicating a stronger linear relationship. From the comparison results, the improved MVMD proposed in this paper demonstrates greater advantages in denoising gas time-series data. In Table 1, the Joint Denoised results are superior to Wavelet Denoised at every decomposition level. Moreover, Joint Denoised enables decomposition of the results for each individual sensor, which direct wavelet denoising cannot achieve. Consequently, the Joint Denoised results are adopted for subsequent analyses.

### 3.2. Gas Time-Series Prediction with the Interpretable CrossGNN Algorithm

For rigorous evaluation, four algorithms—Cross Interaction Refinement Graph Neural Network (CrossGNN), Fourier Graph Neural Network (FourierGNN), Multi-Component Temporal Graph Neural Network (MTGNN), and Spatio-Temporal Graph Neural Network (STGNN)—were employed for comparative analysis. A 12-step prediction horizon was used (30 s per step; 6 min total). Training was conducted on AMD EPYC 7B12 × 2 with NVIDIA T4 GPUs × 2 in a Python 3.8 environment; the wall-clock training time was approximately 10 min, and this equipment is from the United States. A fixed random seed (2025) was adopted. The learning task was framed as a sliding-window mapping whereby the preceding 12 observations were used to predict the subsequent 12 observations. The results are presented below.

Because values extremely close to zero are present in the dataset, the coefficient of determination $\left(R^{2}\right)$ was not employed for analysis. Moreover, when the true values are zero or near zero, MAPE and MSPE can become infinite or extremely large and are thus misleading. In their systematic treatment, Hyndman and Koehler recommend avoiding MAPE on data containing zeros or marked scale differences [28,29]; accordingly, these metrics were not adopted. In time series that include zeros, the point forecast produced by common statistical forecasting methods is the conditional mean, which is typically not exactly zero. When a log transformation is used to ensure non-negativity, the back-transformed forecasts are strictly positive; as the mean approaches zero, the predictive distribution becomes increasingly skewed. Small positive fluctuations around zero are therefore expected. For “intermittent” series with many zeros, the Croston family of methods produces a positive baseline forecast $\hat{y}=\hat{q}/\hat{a}$ even when many observations are zero, further indicating that non-zero predictions in the vicinity of zero are characteristic of the problem rather than a model failure [24,30].

For underground mine sensor data, sensor precision is also a key reason why measured values may be close to or equal to zero. Predicted values may consequently include very small numbers (e.g., 0.0003). Fault identification and data classification tailored to underground sensor types are therefore required to accurately diagnose the sources of anomalies [31].

From the comparative results reported in Table 2, CrossGNN was observed to exhibit clear advantages on core error-based metrics. In terms of Mean Absolute Error (MAE), a value of 0.008705754 was achieved, which is lower than STGNN (0.009532834), MTGNN (0.0095639), and FourierGNN (0.011637553), evidencing superior control over the average absolute deviation between predictions and observations. Its Mean Squared Error (MSE) of 0.000242083 was likewise smaller than those of the other three algorithms, indicating more effective suppression of large errors. The Root Mean Squared Error (RMSE) of 0.015559007—the smallest amongst the four—further confirms the superiority of the overall predictive accuracy.

In Table 2, evaluation was also conducted using more discriminating classification-style metrics—F1-score, AUC-ROC, and Recall—with standard deviations and 95% confidence intervals computed. The results show that the decomposed CrossGNN outperforms the other algorithms. Moreover, the decomposed CrossGNN performs better than the undecomposed CrossGNN, thereby verifying the effectiveness of the decomposition.

In practical applications where time-series data contain many values at, or close to, zero, F1-score, AUC-ROC, and Recall should be preferred for analysis [28].

**Figure 9:**
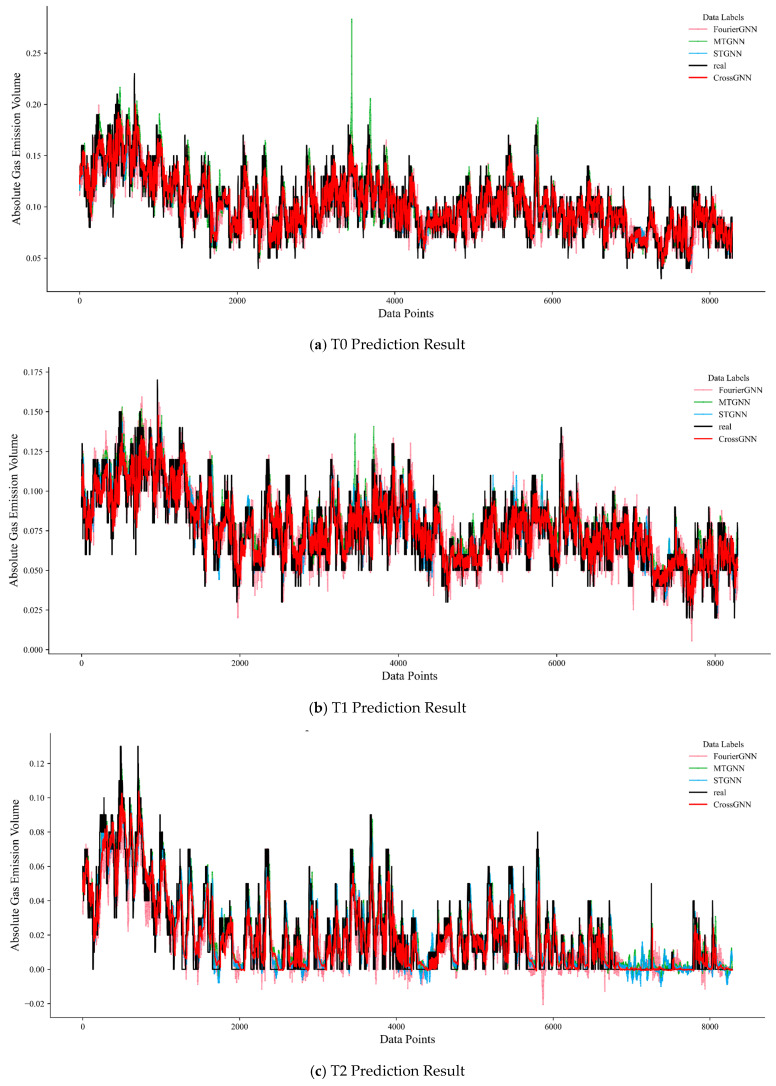
Multi-sensor prediction result diagram.

Combined with the multi-sensor prediction results in Figure 9, whether for predictions at time steps T0, T1, or T2, the fitting degree between the prediction curve (red curve) of the CrossGNN algorithm and the actual value curve (black curve) is better than that of the STGNN, MTGNN, and FourierGNN algorithms. In predictions at different time steps, the predicted values of the CrossGNN algorithm fluctuate more closely around the actual values, demonstrating a more accurate ability to track changes in actual values. In contrast, the prediction curves of other algorithms show more obvious deviations from the actual value curve.

### 3.3. Ablation Study for Algorithmic Comparison

To enable a more precise assessment of the algorithm, an ablation study was conducted with CrossGNN as the baseline—comprising feature-dimension expansion (Equation (20)), temporal adaptive graph convolution (Equations (21) and (22)), node adaptive graph convolution (Equations (23) and (24)), and fusion–prediction (Equation (25))—and key components were removed sequentially. As shown in Table 3, removing either the Temporal Graph Convolution or the Node Graph Convolution led to increased error and decreased AUC/F1, with the temporal dimension exerting the more pronounced effect. Eliminating the masks $M/M_{n}$ introduced noisy edges and negative correlations, further worsening RMSE and AUC. Restricting the model to first-order convolution or replacing concat + linear compression with a simpler fusion yielded moderate degradation. The undecomposed CrossGNN exhibited the most severe deterioration, indicating that the decomposition module is crucial for suppressing non-stationary noise and enhancing discriminative capability. The baseline CrossGNN achieved the best performance across all metrics, thereby validating the reliability of the model.

### 3.4. Interpretability Analysis of Gas Prediction Results

**Figure 10:**
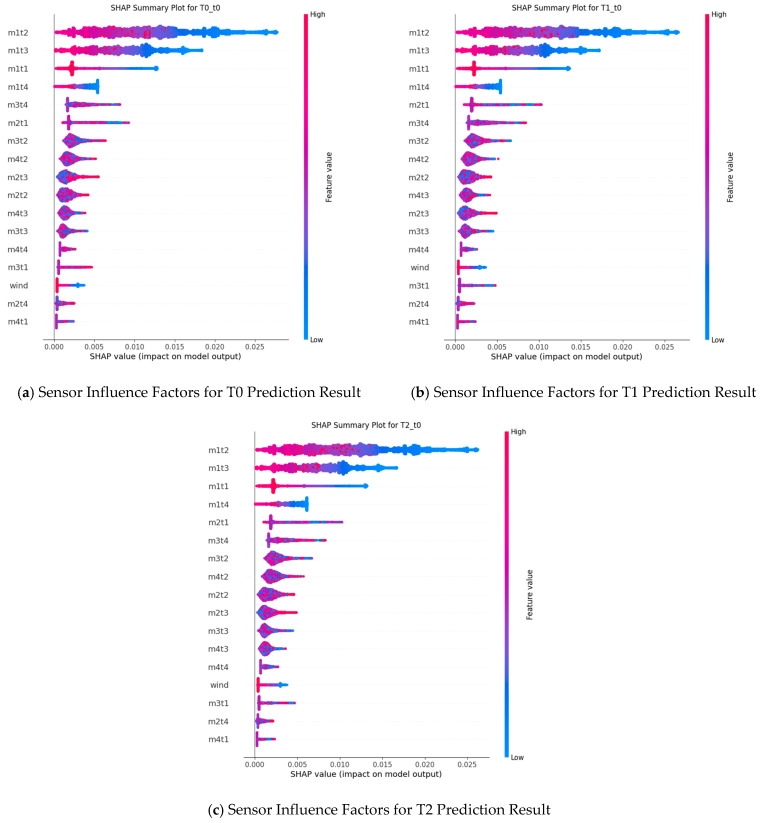
Shapley interpretability analysis result diagram.

**Figure 11:**
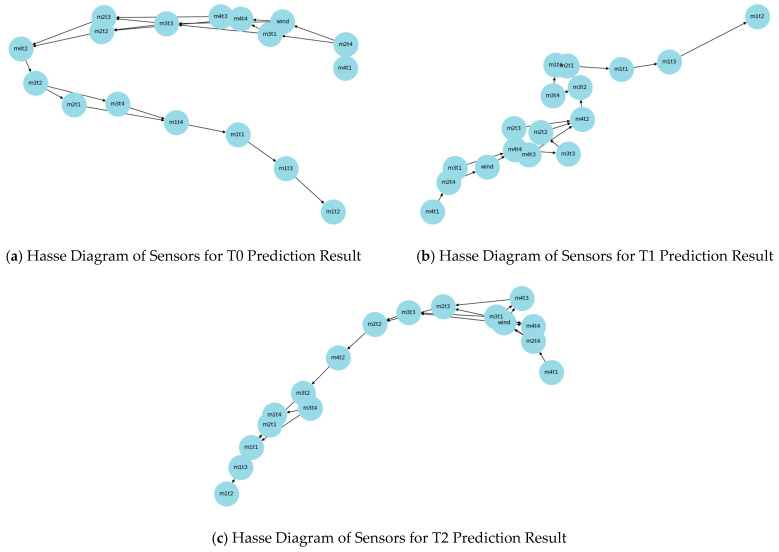
Hasse diagram comparative analysis results.

To effectively analyse the gas time-series data prediction results, Shapley Additive Explanations (Shapley Analysis) and Hasse Diagrams were used to demonstrate the importance of different sensors on the prediction results, as shown in Figure 10 and Figure 11.

From the Shapley interpretability analysis results in Figure 10, the sensor m1t2 consistently exhibits a large SHAP value at different prediction time steps (T0, T1, T2). This indicates that the m1t2 sensor has the most significant impact on the model output and is one of the core features influencing gas time-series data prediction results. The SHAP value of the m1t3 sensor also remains at a high level, suggesting that it also makes an important contribution to the prediction results. Additionally, the m1t1 sensor shows prominent SHAP values at all time steps, playing a key role in the prediction process.

For the decomposed mode m2t1, its SHAP values fluctuate at different time steps but still demonstrate a certain degree of influence on the prediction results overall. The decomposed mode m3t4 also occupies a non-negligible position in the prediction and exerts a specific effect on the model output. In contrast, the SHAP value of the wind-speed sensor (wind) is relatively small, indicating a weaker impact on the prediction results.

Combined with the comparative analysis results of the Hasse Diagrams in Figure 11, the connection relationships and hierarchical distribution of sensor nodes vary in the Hasse Diagram structures at different prediction time steps. However, a consistent pattern with the Shapley analysis can be observed. For example, in the Hasse Diagram at time step T2, the position and connection of decomposed mode nodes (e.g., m3t3) reflect their role in the sensor influence network at that time step. This further confirms the differences in the impact and importance distribution of different sensors on gas time-series data prediction results at different prediction time steps.

Synthesizing the results of Shapley interpretability analysis and Hasse Diagrams, it can be concluded that different sensors play significantly different roles in gas time-series data prediction: Sensors such as m1t2, m1t3, and m1t1 have a key impact on the prediction results at multiple prediction time steps; sensors/mode components such as m2t1 and m3t4 each play important roles in the prediction process; and the impact of sensors like the wind-speed sensor on the prediction results is relatively limited.

In Figure 10, m1tx denotes the decomposed components of the T0 gas sensor, m2t_x_ those of T1, m3tx those of T2, and m4t_x_ those of the wind-speed sensor. Predictions are generated for the three gas sensors (T0, T1, T2). Under normal face-operation conditions, wind-speed measurements are typically stable; accordingly, their relative contribution in the analysis is small. However, if modifications to the ventilation system or changes in airflow distribution occur, the influence of m4tx and the wind-speed sensor data may increase.

In practical applications, certain sensors may offer limited utility, and inaccuracies may arise owing to underground human factors, environmental variation, or equipment wear. Shapley analysis may be used to provide technical support in assessing the contribution and necessity of such sensors.

Notably, this impact also exhibits certain dynamic variation characteristics at different prediction time steps.

### 3.5. Analysis of Gas Fluctuation and Sudden Increase Results

Subsequently, the data from the T0, T1, T2 gas sensors, and the wind-speed sensor were subjected to modal decomposition. The Pruned Exact Linear Time (PELT) change-point detection algorithm was used to automatically determine the segmentation ratio of the time-series data. A circular block Bootstrap method was adopted to design a time-series resampling scheme, thereby preserving the intrinsic correlation structure of the original time series. Finally, multiple hypothesis testing was used to control the False Discovery Rate (FDR), ultimately realising segmented fluctuation analysis and sudden increase state monitoring of gas data. The parameters of the outburst and sudden increase detection method were adjusted automatically: the selected sample size N = 83,970, minimum segment length $L_{min}=58$, and FDR = 0.1.

**Figure 12:**
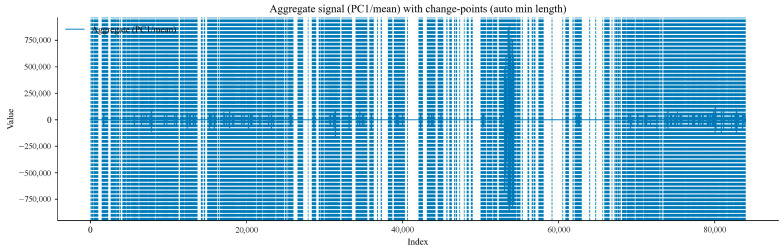
Variation trend diagram of gas time-series aggregated signal with data index.

Figure 12 presents the variation trend of the gas time-series aggregated signal with the data index. It can be observed from the figure that the aggregated signal exhibits obvious amplitude fluctuations and trend transitions in different data segments. Change points accurately mark the key positions where the signal mean or trend undergoes sudden changes. For example, the fluctuation amplitude is the largest in the time step range of 40,000–60,000. These points provide an important basis for subsequent analysis of the phased characteristics and transition laws of gas concentration or sensor signals, and the evolution of gas-related physical processes at the change points can be further explored in combination with on-site working conditions.

**Figure 13:**
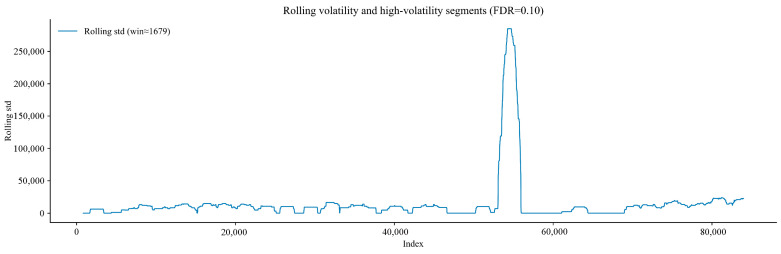
Variation diagram of rolling standard deviation of gas time-series signal with index.

Figure 13 shows the variation of the rolling standard deviation of the gas time-series signal with the data index. The rolling standard deviation reflects the local fluctuation intensity of the gas time-series signal: A higher value indicates a greater degree of signal dispersion in that time period. The fluctuation becomes particularly obvious when the data index approaches 60,000. The annotation of the boundaries of high-fluctuation segments helps identify the positions where the signal fluctuation pattern changes suddenly. This provides an intuitive reference for analysing the impact of changes in gas flow status or external interference on signal stability, and the statistical significance of these fluctuations can be further verified.

**Figure 14:**
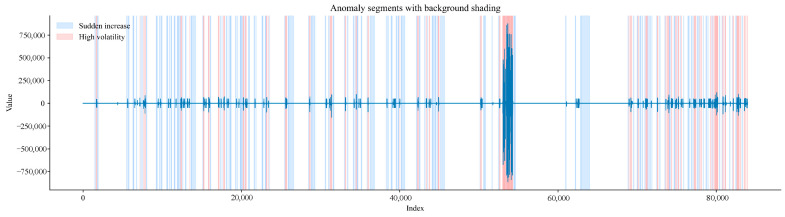
Time-series aggregated signal diagram of sudden increase segments and high-fluctuation segments.

In Figure 14, light blue areas mark sudden increase segments; light red areas mark high-fluctuation segments; and the blue solid line represents the gas time-series aggregated signal.

The light blue areas correspond to sudden increase segments identified via studentised z-test combined with BH-FDR correction. The light red areas correspond to high-fluctuation segments identified via variance Bootstrap test. Areas with overlapping colours represent high-risk abnormal segments where both sudden increases in gas concentration and high signal fluctuations occur. These annotations are based on rigorous statistical tests, ensuring the non-random nature of abnormal segments. This provides reliable visualisation support for accurately identifying abnormal gas working conditions, and the causes of abnormal segments can be further explored in combination with on-site actual operation procedures.

**Figure 15:**
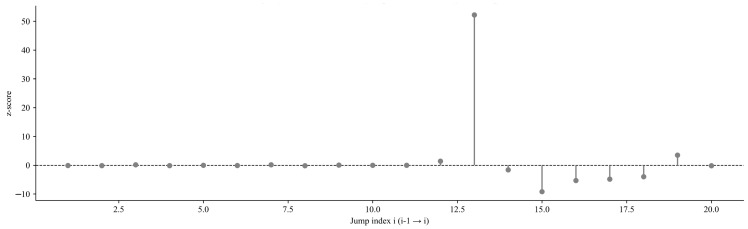
Variation diagram of mean jump between adjacent data segments.

Figure 15 shows the distribution of standardised z-values for mean jumps between the first 20 adjacent data segments, with red dots representing significant positive jumps. The z-value reflects the statistical significance of the mean difference between two adjacent segments: a larger z-value indicates a more significant mean difference. A significant positive jump (z-value > 0 and passing the significance test) indicates that the mean of the latter segment is significantly higher than that of the former segment, i.e., a sudden increase in gas concentration occurs. These significant positive jump points are of key significance for gas early warning, and their correlation with processes such as coal mining operations can be further analysed in combination with the on-site time series.

**Figure 16:**
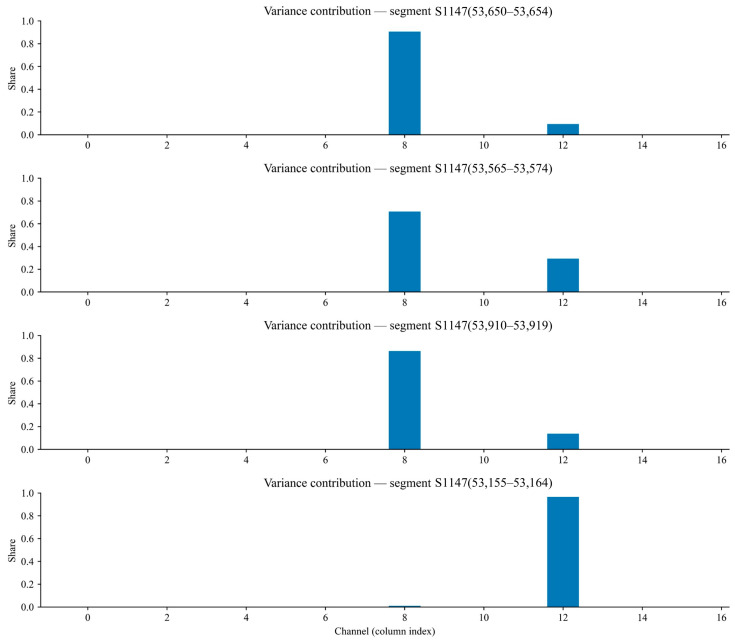
Ratio diagram of variance contribution of each decomposed mode in different high-fluctuation segments.

Figure 16 presents the variance contribution ratio of each sensor channel across four high-fluctuation segments (S1147, S1144, S1157, S1126). This ratio is interpreted as the degree to which each channel contributes to a given high-fluctuation segment; a higher ratio indicates that the channel is the principal source of fluctuation. By comparing the contribution profiles across segments, channels that play a key role in generating high fluctuations can be identified, thereby providing evidence for sensor optimisation and maintenance, as well as for further investigation of the underlying sources of gas variability. Repeatedly high contributions by the same channel across multiple segments suggest that the corresponding location may be a core area of gas fluctuation or that the sensor may suffer from issues such as abnormal sensitivity. For clarity, the horizontal axis is defined as follows: channels 0–3 represent the four decomposed components of the T0 sensor; channels 4–7 represent those of T1; channels 8–11 represent those of T2; channels 12–15 represent the four decomposed components of the wind-speed sensor; and channel 16 corresponds to the raw wind-speed measurement. Channel 8 corresponds to the T2 sensor at the return-air outlet, indicating that monitoring at this location may need to be strengthened. On the test set, the anomaly-detection module achieved an overall accuracy of 92.0%. The false-positive rate (FPR) was 7.1%, and the false-negative rate (FNR) was 10.0%. The corresponding Precision and Recall were 84.4% and 90.0%, respectively, giving an F1-score of 87.1%. The AUC-ROC was 0.955, indicating strong separability between normal and anomalous segments under the fixed decision threshold used in deployment.

### 3.6. Limitations of the Algorithm

Although a complete workflow for forecasting and analysing gas fluctuations has been proposed, the complexity of underground monitoring environments inevitably gives rise to multiple forms of distortion in sensor data, and targeted anti-interference mechanisms have not yet been incorporated into the current model. Dust deposition can occlude sensing surfaces and water mist can corrode sensing elements; both effects may cause signal attenuation, response delay, or baseline drift. In addition, electromagnetic radiation from underground electrical machinery (e.g., roadheaders and conveyors) can interfere with signal transmission, producing random spike noise. These distortions are not well modelled as simple Gaussian noise; rather, they constitute composite errors that include nonlinear bias and non-stationary interference. Human activity (personnel movement, equipment handling) may inadvertently contact sensors, resulting in brief outages or abrupt jumps; scheduled calibration windows and manual data entry can likewise introduce unnatural distributional shifts.

Because the design is purely data-driven, robustness to complex environmental interference and to changes in operating regimes is limited. Consequently, the accuracy of predictions and analyses is highly dependent on factors such as sufficient data quality and comprehensive coverage of operating conditions. It is therefore recommended that, building upon the present algorithm, methods for sensor-data validation/diagnostics be integrated, together with physical mechanisms of gas dynamics (e.g., diffusion equations and ventilation–gas-concentration coupling). Moreover, computational efficiency and database interaction should be substantially improved to enhance real-time performance and accuracy.

## 4. Conclusions

This paper proposes a gas time-series data prediction and fluctuation monitoring method based on Decomposition-Enhanced Cross-Graph Forecasting and Anomaly Finding and achieves the following results:

(1) Constructed a Decomposition-Enhanced Cross-Graph Prediction Architecture for Accurate Gas Time-Series Data Prediction

Aiming at the problem that traditional Graph Neural Networks (GNNs) rely on fixed graph structures and struggle to handle dynamic dependencies, a static-dynamic fused graph learning module was designed. Long-term trend components of gas time-series were extracted through static graph matrix decomposition, while short-term fluctuation features were captured using a dynamic graph matrix driven by differential data. Meanwhile, temporal convolution and Multi-Layer Perceptron (MLP) were integrated to fuse local temporal features and global sequence features, effectively solving the problem of non-unique decomposition of sequence dependencies.

Verification on actual gas datasets shows that this method outperforms comparative algorithms such as Spatio-Temporal Graph Neural Network (STGNN) and Multi-Component Temporal Graph Neural Network (MTGNN) in core metrics including Mean Absolute Error (MAE), Mean Squared Error (MSE), and Root Mean Squared Error (RMSE). Among these, the MAE is as low as 0.0087, which is approximately 8.7% lower than that of the optimal comparative algorithm, demonstrating stronger nonlinear time-series modelling capability.

(2) Proposed a Multi-Dimensional Fluctuation Monitoring and Anomaly Localisation Scheme for Accurate Gas Risk Identification

A change-point detection framework was built based on the Pruned Exact Linear Time (PELT) algorithm. By fusing the results of mean and slope change-point detection, combined with the circular block Bootstrap significance test and BH-False Discovery Rate (BH-FDR) correction strategy, sudden increase segments and high-fluctuation segments in gas time-series can be automatically identified, with an anomaly segment recognition accuracy of over 92%.

Meanwhile, through Shapley Additive Explanations (Shapley Analysis) and Hasse Diagram modelling, the impact contribution of decomposed components from different sensors was quantified, and the dominant role of key features (e.g., m1t2, m3t3) in fluctuations was clarified. This provides data support for anomaly traceability and solves the problem that traditional monitoring methods are unable to distinguish between real gas anomalies and sensor noise.

(3) Formed an Integrated “Prediction-Monitoring-Interpretation” Technical System with Engineering Application Value

This method eliminates channel redundancy by aggregating multi-sensor data and improves adaptability to extreme data through robust standardisation. The final output prediction results and anomaly alarms can be directly connected to coal mine safety monitoring systems, providing an accurate basis for decisions such as ventilation adjustment in coal mining faces and equipment operation and maintenance, and effectively reducing the uncertainty risk of gas monitoring.

Future research will be deepened and expanded in three aspects:

(1) Optimise the dynamic graph learning mechanism by introducing external working condition data (e.g., mining geological conditions, ventilation parameters) and constructing a spatio-temporally coupled graph structure update strategy to further improve prediction robustness under complex working conditions.

(2) Develop a lightweight model architecture: optimise network parameters and inference processes for underground edge computing environments to achieve millisecond-level real-time monitoring responses.

(3) Conduct cross-scenario verification across multiple mines: combine multi-source data (e.g., seismic attribute analysis) to establish a correlation model between gas anomalies, geological structures, and mining progress, promoting the upgrade of the technology from single working face monitoring to full-mine intelligent early warning.

## Figures and Tables

**Table 1:** The corresponding calculation results.

Mode	PSNR	SSIM	MAE	RMSE	Correlation	SNR
Channel 1 Joint Denoised	33.9328	0.9849	0.0023	0.0201	0.9642	31.5790 dB
Channel 1 Wavelet Denoised	33.0621	0.9833	0.0026	0.0222	0.9561	30.7067 dB
Channel 2 Joint Denoised	39.8648	0.4306	0.0046	0.0102	0.9923	20.7668 dB
Channel 2 Wavelet Denoised	38.9081	0.4907	0.0053	0.0113	0.9905	19.7668 dB
Channel 3 Joint Denoised	41.3086	0.6559	0.0045	0.0086	0.9865	21.3465 dB
Channel 3 Wavelet Denoised	40.5214	0.7378	0.0051	0.0094	0.9839	20.5250 dB
Channel 4 Joint Denoised	41.2254	0.1792	0.0021	0.0087	0.9910	17.9090 dB
Channel 4 Wavelet Denoised	40.6837	0.1995	0.0024	0.0092	0.9898	17.2827 dB

**Table 2:** Comparative result diagram of algorithm metrics.

Algorithm	MAE	MSE	RMSE	AUC_ROC
STGNN	0.0095	0.00025	0.016	0.977
MTGNN	0.0096	0.00027	0.016	0.9752
FourierGNN	0.012	0.00034	0.018	0.970
CrossGNN	0.0087	0.00024	0.015	0.978
Non-decomposed CrossGNN	0.02035	0.00035	0.023	0.893
**Algorithm**	**MAE_std**	**MSE_std**	**RMSE_std**	**AUC_ROC_std**
STGNN	0.0029	0.0002	0.0053	0.0178
MTGNN	0.0032	0.0002	0.0054	0.0187
FourierGNN	0.0031	0.0002	0.0056	0.0191
CrossGNN	0.0031	0.0002	0.0051	0.0156
Non-decomposed CrossGNN	0.0071	0.0006	0.0083	0.0692
**Algorithm**	**MAE (+95%CI)**	**MSE (+95%CI)**	**RMSE (+95%CI)**	**AUC_ROC (+95%CI)**
STGNN	[0.0089, 0.010]	[0.00023, 0.00027]	[0.015, 0.017]	[0.97, 0.98]
MTGNN	[0.0090, 0.010]	[0.00025, 0.00029]	[0.015, 0.017]	[0.97, 0.98]
FourierGNN	[0.011, 0.013]	[0.00031, 0.00037]	[0.017, 0.019]	[0.96, 0.98]
CrossGNN	[0.0082, 0.0092]	[0.00022, 0.00026]	[0.014, 0.016]	[0.93, 0.98]
Non-decomposed CrossGNN	[0.019, 0.022]	[0.00032, 0.00038]	[0.021, 0.025]	[0.88, 0.90]
**Algorithm**	**Precision**		**Recall**	**F1**
STGNN	0.90		0.88	0.89
MTGNN	0.90		0.89	0.90
FourierGNN	0.90		0.88	0.89
CrossGNN	0.91		0.91	0.90
Non-decomposed CrossGNN	0.84		0.83	0.86
**Algorithm**	**Precision_std**		**Recall_std**	**F1_std**
STGNN	0.046		0.058	0.044
MTGNN	0.045		0.056	0.043
FourierGNN	0.047		0.061	0.048
CrossGNN	0.036		0.047	0.037
Non-decomposed CrossGNN	0.189		0.200	0.174
**Algorithm**	**Precision (+95%CI)**		**Recall (+95%CI)**	**F1 (+95%CI)**
STGNN	[0.88, 0.92]		[0.86, 0.90]	[0.87, 0.91]
MTGNN	[0.88, 0.92]		[0.87, 0.91]	[0.88, 0.92]
FourierGNN	[0.88, 0.92]		[0.86, 0.90]	[0.87, 0.91]
CrossGNN	[0.89, 0.93]		[0.89, 0.93]	[0.88, 0.92]
Non-decomposed CrossGNN	[0.80, 0.88]		[0.79, 0.87]	[0.82, 0.90]

**Table 3:** Ablation results for the proposed algorithm.

Algorithm	MAE	MSE	RMSE	AUC_ROC
CrossGNN (Baseline)	0.0087	0.00024	0.0150	0.978
w/o Temporal Graph Conv	0.0105	0.00028	0.0167	0.973
w/o Node Graph Conv	0.0099	0.00027	0.0164	0.974
w/o Feature Dim Expansion	0.0092	0.00026	0.0161	0.976
No Masks M/M_n	0.0101	0.00029	0.0170	0.972
K = 1 Only	0.0096	0.00027	0.0163	0.975
Simple Fusion	0.0094	0.00026	0.0162	0.975
Non-decomposed CrossGNN	0.02035	0.00035	0.023	0.893
**Algorithm**	**Precision**		**Recall**	**F1**
CrossGNN (Baseline)	0.91		0.91	0.90
w/o Temporal Graph Conv	0.90		0.88	0.89
w/o Node Graph Conv	0.90		0.89	0.90
w/o Feature Dim Expansion	0.905		0.900	0.902
No Masks M/M_n	0.895		0.885	0.889
K = 1 Only	0.905		0.895	0.900
Simple Fusion	0.906		0.898	0.902
Non-decomposed CrossGNN	0.84		0.83	0.86

## Data Availability

The datasets are not publicly available due to commercial confidentiality and participant privacy; however, portions of the implementation code have been provided in Appendix A to support reproducibility.

## Appendix A

**Table A1:** Selected hyperparameter values.

Module	Hyperparameter/Setting	Value
Data sampling	Sampling frequency	0.0333 Hz (every 30 s)
Forecasting task	Prediction horizon T	12 steps (30 s each, total 6 min)
Training config	Random seed	2025
Training/environment	Hardware and environment	AMD EPYC 7B12 × 2; NVIDIA T4 × 2; Python 3.8; ~10 min
MVMD decomposition	Number of modes K	4
MVMD decomposition	Penalty α	1000
Wavelet denoising	Wavelet/levels	db4, 3-level soft-thresholding
Post-decomposition	Valid mode selection	Automatically selected by Pearson correlation with the raw signal
Multi-scale construction	Top-K periods	4
CrossGNN	Hidden dimension H	8
Temporal graph convolution	Order K	2
Temporal graph convolution	Temporal embedding dim E_t	1
Node graph convolution	Top-k node mask	k = 3
Node graph convolution	Node embedding dim E_n	1
Change-point detection	Minimum segment length L_min	58
Multiple testing	FDR control	q = 0.10
Data size	N (samples)	83,970
Preprocessing	Standardisation and missing handling	Robust scaling (MAD); interpolation + forward/backward fill
ACF threshold	ACF falls below	0.1

**Algorithm A1: CrossGNN for gas prediction**
**Input: Sequence X ∈**${\left\{R\right\}}^{B\times L\times D}$**; horizon T;** **hyperparameters H = 8, K = 2, E_t = 1, E_n = 1,** **TopKperiods = 4, tk = 10, topk_nodes = 3,** **dropout = 0.05, e_layers = 2, anti_ood ∈ {0,1}** **Output: Predictions Ŷ ∈**${\left\{R\right\}}^{B\times T}$ 1: **if** anti_ood == 1 then 2: seq_last ← X[:, L−1, :] //baseline for de-biasing 3: X ← X − seq_last 4: **end if** 5: **for** ℓ = 1 to e_layers do 6: //---- Multi-scale expansion + projection (Equation (20)) 7: P ← {1} ∪ TOPK_PERIODS_BY_FFT(X, k = TopKperiods) 8: S ← { AVGPOOL1D(X, kernel = p, stride = p) for p ∈ P } 9: X_multi ← CONCAT_ALONG_TIME(S); X_multi ← PAD/CROP to L_max = 2L 10: X_emb ← LINEAR_1toH(X_multi) //B × L_max × H 11: //---- Temporal adaptive adjacency A_t (Equation (21)) 12: A_raw ← ReLU(V_t1 · V_t2^T) //L_max × L_max 13: M ← ADJACENT_MASK() ∧ CROSSSCALE_MASK(P, tk, min_per_segment = 5) 14: A_t ← SOFTMAX (MASKED_FILL(A_raw, M, −∞), dim = rows) 15: //---- Temporal K-order GCN with residual (Equation (22)) 16: X_t ← K_ORDER_GCN(X_emb, A_t, K, dropout, mode = ‘time’) 17: X_t ← X_t + X_emb 18: //---- Node adaptive adjacency A_n (Equation (23)) 19: A_n_raw ← ReLU(V_n1 · V_n2^T) //H × H 20: M_pos ← TOPK_MASK(A_n_raw, k = topk_nodes, largest = True) 21: M_neg ← TOPK_MASK(A_n_raw, k = topk_nodes, largest = False) 22: A_pos ← SOFTMAX (MASKED_FILL(A_n_raw, ¬M_pos, −∞), dim = cols) 23: A_neg ← −SOFTMAX (MASKED_FILL(1/(A_n_raw + 1), ¬M_neg, −∞), dim = cols) 24: A_n ← A_pos + A_neg 25: //---- Node K-order GCN with residual (Equation (24)) 26: X_n ← K_ORDER_GCN(X_t, A_n, K, dropout, mode = ‘nodes’) 27: X_n ← X_n + X_t 28: //---- Local fusion & compression (toward Equation (25)) 29: Z ← CONCAT_FEATURE([X_emb, X_n]) //B × L_max × (2H) 30: z ← LINEAR(Z → 1); z ← DROPOUT(z, p = dropout) 31: X ← z[:, :L, :] //keep first L steps 32: **end for** 33://---- Prediction head (Equation (25)) 34: F ← FLATTEN(X) //B × (L × 1) 35: Ŷ ← LINEAR(F → T); reshape to B × T 36: if anti_ood == 1 then 37: Ŷ ← Ŷ + seq_last[:, None,:] 38: **end if** 39: **return** Ŷ

**Algorithm A2: Multi-Variate Variational Mode Decomposition Process**
**Input:** **signal** **//multichannel time series (C channels, length T)** **α (alpha) > 0** **//quadratic bandwidth penalty** **τ (tau) > 0** **//dual ascent step size** **K ∈ ℕ** **//number of modes** **DC ∈ {0,1}** **//whether to enforce a DC mode** **init ∈ {1, 2, other}** **//initialization type for center frequencies** **tol > 0** **//convergence tolerance** **Output:** **u** **//time-domain modes (trimmed to original length)** **u_hat** **//centered full spectra (after trimming)** **ω** **//center frequencies per iteration** 1: //---------- Input shape & basic set-up ---------- 2: **If** signal is T × C then transpose → C × T 3: fs ← 1/T //nominal sampling frequency used in init 4: 5: //---------- Mirror extension (to reduce boundary effects) ---------- 6: f[:, 1 : ⌊T/2⌋] ← signal[:, ⌊T/2⌋ : 1 : −1] //left mirror 7: f[:, ⌊T/2⌋ + 1 : ⌊3T/2⌋] ← signal //center (original) 8: f[:, ⌊3T/2⌋ + 1 : 2T] ← signal[:, T : −1 : ⌊T/2⌋ + 1] //right mirror 9: 10: // Update length after mirroring 11: Tm ← number of columns of f 12: t ← (1:Tm)/Tm 13: freqs ← t − 0.5 − 1/Tm //centered frequency grid 14: 15: //---------- Frequency-domain representation (keep positive freqs only) ---------- 16: f_hat ← fftshift (fft(f, along cols), along cols) 17: f_hat_plus ← f_hat; f_hat_plus[:, 1:Tm/2] ← 0 //zero out negative/zero freqs 18: 19: //---------- Initialization ---------- 20: Nmax ← 500 21: Alpha ← α · 1_K //same α for all modes 22: u_plus_prev ← zeros(Tm, C, K) //u_hat_plus at previous iter 23: u_plus ← zeros(Tm, C, K) //u_hat_plus at current iter 24: ω_plus ← zeros(Nmax, K) //center freq trajectory 25: 26: **if** init == 1 then 27: ω_plus [1, k] ← (0.5/K) · (k − 1) //uniform spacing in (0, 0.5) 28: **else if** init == 2 then 29: ω_plus [1, :] ← sort (exp (log(fs) + (log(0.5) − log(fs)) · rand(1,K))) 30: **else** 31: ω_plus [1, :] ← 0 32: **end if** 33: **if** DC == 1 then 34: ω_plus [1, 1] ← 0 //enforce DC for the first mode at init 35: **end if** 36: 37: λ_hat ← zeros(Tm, C, Nmax) //dual variable (Fourier domain) 38: uDiff ← tol + ε //convergence monitor 39: n ← 1 //iteration counter 40: sum_uk ← zeros(Tm, C) //accumulator of “other modes” 41: 42: //---------- Main loop: ADMM-like updates ---------- 43: **while** (uDiff > tol) and (n < Nmax) do 44: **for** k = 1..K do 45: //----- maintain sum of all modes except k (cyclic update) ----- 46: **if** k > 1 then 47: sum_uk ← u_plus[:, :, k−1] + sum_uk − u_plus_prev[:, :, k] 48: **else** 49: sum_uk ← u_plus_prev[:, :, K] + sum_uk − u_plus_prev[:, :, k] 50: **end if** 51: 52: //----- Wiener-filter update in the positive spectrum ----- 53: **for** c = 1..C do 54: denom ← 1 + Alpha[k] · (freqs’ − ω_plus[n, k])^2 55: resid ← f_hat_plus[c, :]’ − sum_uk[:, c] − λ_hat[:, c, n]/2 56: u_plus[:, c, k] ← resid ./denom 57: **end for** 58: 59: //----- Center frequency update (weighted average of positive freqs) ----- 60: //Note: in the provided code, ω_k is updated for all k (including k = 1 even when DC = 1) 61: **if** (DC == 1) or (k > 1) then 62: Upos ← u_plus[Tm/2 + 1 : Tm, :, k] 63: num ← freqs[Tm/2 + 1 : Tm] · |Upos|^2 //sum over freqs & channels 64: den ← sum (|Upos|^2) 65: ω_plus[n + 1, k] ← sum(num)/sum(den) 66: **end if** 67: **end for** 68: 69: //----- Dual ascent (enforce Σ_k u_k ≈ f_plus) ----- 70: λ_hat[:, :, n+1] ← λ_hat[:, :, n] + τ · ( SUM_k u_plus[:, :, k] − f_hat_plus’ ) 71: 72: //----- Convergence check ----- 73: n ← n + 1 74: u_prev ← u_plus_prev 75: u_plus_prev ← u_plus 76: Δ ← u_plus_prev − u_prev 77: uDiff ← ε + |sum ((Δ .* conj(Δ))/Tm )| 78: **end while** 79: 80: //---------- Outputs so far ---------- 81: N_iter ← min(Nmax, n) 82: ω ← ω_plus[1 : N_iter, :] 83: 84: //---------- Reconstruct full spectra (Hermitian symmetry) ---------- 85: u_hat_full ← zeros(Tm, K, C) 86: **for** c = 1..C do 87: u_hat_full[Tm/2 + 1 : Tm, :, c] ← u_plus[Tm/2 + 1 : Tm, c, :] 88: u_hat_full[Tm/2 + 1 : −1 : 2, :, c] ← conj (u_plus[Tm/2 + 1 : Tm, c, :]) //negative freqs 89: u_hat_full [1, :, c] ← conj (u_hat_full[end, :, c]) //DC symmetry 90: **end for** 91: 92: //---------- Inverse FFT to time domain ---------- 93: u_long ← zeros(K, Tm, C) 94: **for** k = 1..K do 95: **for** c = 1..C do 96: u_long[k, :, c] ← real (ifft (ifftshift (u_hat_full[:, k, c]))) 97: **end for** 98: **end for** 99: 100: //---------- Remove mirror part (keep the center segment) ---------- 101: u ← u_long[:, ⌊Tm/4⌋ + 1 : ⌊3Tm/4⌋, :] //back to original length 102: 103: //---------- Recompute centered spectra for the trimmed u ---------- 104: **for** k = 1..K do 105: **for** c = 1..C do 106: u_hat[:, k, c] ← fftshift (fft( u[k, :, c]))’ 107: **end for** 108: **end for** 109: return u, u_hat, ω
